# Choosing between Internet-based psychodynamic versus cognitive behavioral therapy for depression: a pilot preference study

**DOI:** 10.1186/1471-244X-13-268

**Published:** 2013-10-18

**Authors:** Robert Johansson, Anna Nyblom, Per Carlbring, Pim Cuijpers, Gerhard Andersson

**Affiliations:** 1 Department of Behavioural Sciences and Learning, Linköping University, Linköping, Sweden; 2 Department of Psychology, Stockholm University, Stockholm, Sweden; 3 Department of Clinical Psychology and EMGO Institute, Vrije Universiteit, Amsterdam, The Netherlands; 4 Department of Clinical Neuroscience, Psychiatry Section, Karolinska Institutet, Stockholm, Sweden

**Keywords:** Depression, Psychodynamic therapy, Cognitive behavioral therapy, Internet-based psychotherapy, Treatment preferences

## Abstract

**Background:**

Major depression is a world-wide problem that can be treated with various forms of psychotherapy. There is strong research support for treating major depression using cognitive behavior therapy delivered in the format of guided self-help via the Internet (ICBT). Recent research also suggests that psychodynamic psychotherapy can be delivered as guided self-help via the Internet (IPDT) and that it seem to be as effective as ICBT for mild to moderate depression. However, no head-to-head comparison between the two treatments exists. In the field of Internet interventions it is largely unexplored if treatment preference affects outcome and adherence.

**Methods:**

Participants were allocated to IPDT or ICBT based on their stated preference. More than half of the participants preferred ICBT (*N* = 30) over IPDT (*N* = 14). Differences in efficacy between treatments were explored. Correlations between strength of preference and treatment outcome, adherence to treatment and completion of the whole treatment program were explored. Data were collected before and after treatment, as well as in a 7-month follow-up.

**Results:**

During the treatment period, both programs performed equally well in reducing symptoms. More participants who received IPDT completed the entire program. At follow-up, mixed-effects models showed that participants who chose ICBT improved more in terms of quality of life. The ICBT group also had a significant increase in participants who recovered from their depression from post-treatment to follow-up. Exploratory analyses indicated that strength of preference was correlated with adherence to treatment and completion of the whole program, and long-term outcome for the ICBT group.

**Conclusions:**

Few differences were found during the acute treatment phase, but the long-term effects are in favor of ICBT. Strength of preference for treatment seems to have a predictive value. Further research comparing the efficacy of ICBT and IPDT, and the effects of preference matching and strength of preference, is warranted.

**Trial registration:**

This trial is a continuation of the study registered as NCT01324050 at Clinicaltrials.gov.

## Background

Major depression is a common major health problem, which significantly affect the individual and generates substantial costs for society [1,2]. Only 18-25% of patients in a US 12-month prevalence study were adequately treated, indicating that individuals with depression are an undertreated group [1]. Several forms of psychological treatments are effective in the treatment of depression [3]. A majority of studies have examined cognitive behavior therapy (CBT) which has gained a strong research support [3]. Psychodynamic psychotherapy (PDT) is also effective in the treatment of depression [4].

In the last decade, Internet-based psychological treatments have been developed for a range of psychiatric conditions, including depression [5,6]. Research has shown that guided Internet treatments are effective in the treatment of depression and anxiety disorders [7,8]. Close to all of these treatments has been based on cognitive behavior therapy (ICBT) [6]. However, results from a recent randomized trial indicate that guided self-help via the Internet is a promising treatment format also for psychodynamic therapy (IPDT), at least when targeting depression [9], and potentially also for generalized anxiety disorder [10]. While the study by Johansson et al. [9] indicated that IPDT may be on par with ICBT in the treatment of depression, to the best of our knowledge, no head-to-head comparisons exist.

### Patients’ preferences

Patients’ preferences in psychotherapy and pharmacotherapy are generally considered to be a factor that influences treatment outcome and probability of dropout [11,12]. Most studies have investigated the beneficial effect of being matched with one’s preferred treatment related to outcome. More specifically, other studies have investigated the differences in outcome between participants who did receive a preferred treatment and those who did not. Overall, a recent meta-analysis [12] has found this effect size to be *d* = 0.31 (95% CI: 0.20 – 0.43). The analysis found significant heterogeneity among studies, with indications that factors such as study design and type of condition treated may moderate outcome. Moreover, Swift and colleagues [12] found an indication that the effect of being matched to a preferred treatment may be larger in studies investigating psychotherapy vs. pharmacotherapy than in psychotherapy vs. psychotherapy trials.

There is also a substantial number of studies investigating the effect of being matched with preferred treatment, related to treatment dropout. In terms of odds ratios, Swift et al. [12] found the overall effect of preference on dropout to be *OR* = 0.59 (95% CI: 0.44 – 0.78), indicating that the odds of dropping out of therapy prematurely in participants who were matched to their preference were between a half and a third, compared to participants who were not matched.

While many studies exist on the effect of preference matching, there are, to our knowledge, only two studies that investigate the influence of strength of preference on treatment outcome and dropout [13,14]. In Raue et al. [13] participants were asked the question “I wish to receive counseling or psychotherapy for my depression” and strength of preference was measured by a 5-point Likert scale (1, strongly disagree; 5, strongly agree). Raue et al. [13] found that adherence to treatment could be predicted by preference strength but not from preference match in a sample of depressed participants, receiving either 12 weeks of interpersonal psychotherapy plus additional booster sessions, or 20 weeks of escitalopram. Furthermore, strength of preference could predict whether a patient started treatment or not [13]. However, neither preference match nor preference strength could predict symptom remission, although an unexpected negative association was found between preference strength and symptom severity at 12 weeks [13]. Dunlop et al. [14] also investigated the effect of preference strength in addition to preference matching in a study comparing CBT and escitalopram among patients with depression. In contrast to Raue et al. [13], Dunlop et al. [14] did not find any predictive value in strength of preference.

Not much is known regarding the role of preferences in ICBT and IPDT. However, in a pilot study on tailored Internet treatment for anxiety it was found that the treatment was effective when participants were allowed to choose treatment components [15].

In summary, there is some evidence suggesting that being matched to one’s preferred treatment can affect outcome and dropout. However, the predictive value of preference strength is largely unknown.

### Study aims

The primary aim of this pilot study was to make a preliminary investigation of differential efficacy between a psychodynamic and a cognitive-behavioral Internet-delivered psychological treatment in a sample of participants with major depression. A further aim of the study was to test the effect of preference strength on treatment outcome, adherence and completion.

## Methods

### Ethics statement

The study was approved by the Regional Ethics Board of Linköping, Sweden (Registration number 2010/386-31). All participants provided signed informed consent via the online treatment platform.

### Participants

A detailed description of the initial recruitment and selection procedures can be found in Johansson et al. [9]. Briefly, participants were all at least 18 years old and had a diagnosis of major depression, with no risk of suicidality and not having other primary disorders that needed different treatments or that could be affected negatively by the treatment. The current pilot study involved those who participated in the control group of the original trial. As described in Johansson et al. [9], the control condition consisted of a structured support intervention given for 10 weeks. Participants in that group improved considerably, as evident by the within-group effect size of *d* = 0.84 on the BDI-II, which was the primary outcome measure [9].

When the original trial was completed, the participants from the control group (from this point on, these individuals are referred to as “the participants”) were about to be crossed over to treatment. Importantly, at time of the original post-treatment measure the participants were given the opportunity to choose between the IPDT protocol tested in Johansson et al. [9] or a previously developed ICBT treatment [16]. Both treatments targeted depression, lasted for 10 weeks and were delivered via the Internet. Of the original 46 participants, 2 stated that they did not want any further treatment. Of the remaining 44, there were more participants who preferred ICBT (*N* = 30; 68.2%) than those preferring IPDT (*N* = 14; 31.8%). A two-tailed binomial test confirmed the difference to be significant (*p* = .023). Demographic data for the 44 participants are presented in Table 1. No significant differences were found between the two groups at baseline on any measure or demographic variable.

**Table 1:** Demographic description of the participants

		**ICBT (*N*** **= 30)**	**IPDT (*N*** **= 14)**
Gender	Female	20 (66.7%)	10 (71.4%)
	Male	10 (33.3%)	4 (28.6%)
Age	Mean (SD)	46.0 (13.1)	44.4 (12.9)
	Min - Max	25 – 72	21 – 65
Marital status	Married or co-habiting	19 (63.3%)	10 (71.4%)
	Other	11 (36.7%)	4 (28.6%)
Educational level	College or university, at least 3 years	24 (80.0%)	9 (64.3%)
	College or university, shorter than 3 years	3 (10.0%)	2 (14.3%)
	Other	3 (10.0%)	3 (21.4%)
Employment status	Employed	26 (86.7%)	10 (71.4%)
	Other	4 (13.3%)	4 (28.6%)
Medication status at intake	Present	5 (16.7%)	4 (28.6%)
	Prior history	8 (26.7%)	4 (28.6%)
	No history	17 (56.7%)	6 (42.9%)
Psychological treatment	Prior history	17 (56.7%)	7 (50.0%)
	No history	13 (43.3%)	7 (50.0%)
Depression at intake	In acute episode	20 (66.7%)	8 (57.1%)
	In partial remission	10 (33.3%)	6 (42.9%)
Comorbidity at intake	Social anxiety disorder	8 (26.7%)	5 (35.7%)
	Generalized anxiety disorder	7 (23.3%)	3 (21.4%)
	Panic disorder	1 (3.3%)	1 (7.1%)
	Obsessive compulsive disorder	1 (3.3%)	0 (0.0%)
	Post-traumatic stress disorder	0 (0.0%)	0 (0.0%)
	Any anxiety disorder	13 (43.3%)	9 (64.3%)
Strength of preference	Mean (SD)	2.90 (0.85)	2.93 (1.07)
	Min – Max	1 – 4	1 – 4

*Note*: ICBT = Internet-based cognitive-behavioral therapy; IPDT = Internet-based psychodynamic therapy.

### Preference statement and strength of preference

Descriptions of the two treatments, which were roughly half a page each, were provided to the participants. This information was similar in structure for both alternatives, and contained the sections “How psychiatric problems are explained”, “Way of working in therapy” and “Internet-based psychodynamic psychotherapy/cognitive behavioral psychotherapy”. Briefly, the description of psychodynamic therapy emphasized the interpersonal nature of psychiatric problems, the use of reflection as a mean of working with the material and the treatment focus on how to reveal unconscious recurring patterns in life. The description of CBT stated that cognitions and behavioral patterns interact to maintain depression and did also explain how the treatment would address both depressive thinking and help the client expose himself/herself to fearful situations systematically. Furthermore, the CBT description mentioned problem registration and homework as important ingredients in the Internet-based CBT treatment. The descriptions of CBT and PDT were based on material from another research project investigating attitudes towards different treatment alternatives (unpublished). In that project, experts on both CBT and PDT confirmed the descriptions to be representative.

All participants needed to state whether they preferred IPDT or ICBT. Importantly, it was clear to all participants that they would receive the treatment they preferred. In addition, all participants had to state the strength for their preference for the chosen treatment. This was assessed with a single question: “How important is this choice for you?”. Possible answers included 1 = “Not important at all (I does not matter what treatment I receive)”, 2=“Not very important”, 3=“Fairly important”, 4=“Very important (it is very much important which treatment I receive)”. In accordance with Raue et al. [13], we considered preference strength to be a continuous measure.

### Outcome measures

The BDI-II was used as the primary outcome measure and was administered at pre-treatment, weekly during treatment, at post-treatment and at a 7-month follow-up. Secondary measures of depression were the self-rated version of the Montgomery-Åsberg Depression Rating Scale (MADRS-S; [17]) and the 9-item Patient Health Questionnaire Depression Scale (PHQ-9; [18]). Measures of anxiety included the Beck Anxiety Inventory (BAI; [19]) and the 7-item Patient Health Questionnaire Generalized Anxiety Disorder Scale (GAD-7; [20]). Moreover, the Quality of Life Inventory (QOLI; [21]) was included as a measure of life satisfaction. All secondary measures were collected pre-treatment, post-treatment and at the 7-month follow-up. The measures were administered via the Internet, which has been shown to be a valid format for questionnaires regarding depression and anxiety [22,23]. At follow-up, a clinical interview was also conducted which aimed at estimating global improvement, measured by the 7-point version of the Clinical Global Impression-Improvement (CGI-I) scale [24]. The CGI-I ranges from −3 (“very much worse”) to 3 (“very much improved”) where a 0 means “neither better nor worse”.

### Treatments

Both treatments lasted for 10 weeks and were given as guided self-help, with participants taking part of text-based modules through a secure online environment. The modules were complemented by therapist support in a medium similar to e-mail. Procedures and character of support were similar to that of many other guided self-help treatments tested [5]. In both treatments, therapists gave feedback on the clients’ experiences and reported work done during the week. The therapists also adminstered the gradual access to the modules. While we did not log the exact therapist time per client, we have assumed that a similar amount of support were given in this study, as compared to the original study in which 13.2 minutes per client and week were reported [9]. The ICBT treatment was 150 pages of text in total, and the IPDT contained 167 pages. Modules were roughly equal in layout, number of illustrations and number of case examples.

### Psychodynamic treatment

The IPDT treatment was based on the book “Make the leap” [25] and contained extra material including a psychodynamic understanding of how depression is developed and maintained. Participating individuals were guided through a program called SUBGAP, which stands for (I) Seeing unconscious patterns that contribute to emotional difficulties, (II) Understanding these patterns, (III) Breaking such unhelpful patterns, and (IV) Guarding Against Patterns and/or relapses in the future. Briefly, the nine treatment modules included (1) Introduction to SUBGAP; (2) Techniques and practice on how to discover one’s own unconscious patterns; (3) Material on how to understand patterns from both a historical perspective and a here-and-now perspective; (4) Techniques on how to break unhelpful patterns; (5) Material on how to minimize the risk of falling back into one’s old and unproductive patterns; (6) Application of the obtained knowledge in working life; (7) Material on how to apply the knowledge about patterns with a focus on personal relationships; (8) A presentation of a models which connects unconscious patterns and depression, and (9) A summary of the treatment and material on how to prevent relapse. A detailed description can be found in [9]. All modules ended with a proposal on how the participants could try out various SUBGAP strategies that had been presented. Participants were encouraged to contact their therapists once a week to describe their experiences from working with the material. These tasks were encouragements for reflections and were in general about writing about experiences, rather than completing typical exercises. The therapists provided feedback on the participants’ experiences and administered the gradual access to the modules.

### Cognitive behavioral treatment

The ICBT treatment used in this study was based on the treatment protocol tested in previous trials [16,26], but with an extra chapter added on anxiety and worry management. The ten treatment modules included (1) Introduction to depression and the treatment (psychoeducation); (2–3) Behavioral activation; (4–5) Cognitive restructuring; (6) Anxiety and worry management; (7) Sleep management; (8–9) Further practice by combining previous modules, and (10) Relapse prevention and maintenance of progress. All modules contained homework, which typically contained various exercises related to the content from the modules. Examples of exercises include completing activity sheets, working with automatic thoughts and handling sleep problems by managing sleep hygiene. Therapists worked similarly in the ICBT condition, as in the IPDT condition. This included giving feedback and administering the gradual feedback to modules.

### Therapists

Four of the original six last-semester M.Sc. clinical psychologist students were also therapists in this pilot trial. All these four therapists had clients from both treatment groups. These therapists had a general training in psychotherapy, i.e. they were not specialized in neither CBT nor PDT. However, they all had clinical training and 16 weeks of internship. In addition, a clinical psychologist trained in CBT and with experience of the ICBT manual, participated as a therapist in the ICBT group. As described above, the role of the therapists were similar for the two groups. Further clinical procedures were similar to those described in [9].

### Statistical analyses

The study had an open design in that no control group was used. However, differences between groups formed on preference were explored. To investigate group differences, interaction effects of group and time were modeled. The continuous outcome variables were analyzed using random intercept models with Maximum Likelihood estimation. These models have the ability to handle missing data and therefore the intention-to-treat principle was adhered to [27]. Data from pre-treatment, post-treatment and the follow-up were analyzed. For the BDI-II, the weekly measures were also included in the model.

To study if strength of preference was correlated with treatment outcome, the BDI-II scores were transformed before the analyses. In line with the recommendations by Steketee and Chambless [28], residual gain scores for the BDI-II at post-treatment and at follow-up were calculated, instead of using crude raw scores. This calculation transforms individual scores relative typical gains made by other study participants having similar pre-treatment scores. The residual gain scores were calculated using the formula [*Z*_*2*_ – (*Z*_*1*_ × *r*_*12*_)], where *Z*_*2*_ is the standardized score for the second time point (post-treatment or follow-up) and *Z*_*1*_ is the standardized score for the first time point (pre-treatment), and *r*_*12*_ is the Pearson correlation between scores at the two occasions [28]. The correlations between strength of preference and residual gain scores (for post-treatment and follow-up) were studied for the entire sample, as well as for the two treatment groups separately.

Recovery after treatment was investigated at post-treatment and at follow-up. In accordance with Johansson et al. [9], recovery was defined as a BDI-II score of ≤ 10. This definition is also in line with previous trials on depression [29,30].

Within- and between-group effect sizes (Cohen’s *d*) were calculated by dividing the differences in means by the pooled standard deviations, as described in Borenstein et al. [31]. The between-group effect sizes can be interpreted as follows: an effect size in the range of 0.20–0.49 is small, while 0.50–0.79 is moderate, and an effect size over 0.80 is large [32].

## Results

### Attrition and adherence

All participants had provided post-treatment data in the original trial, which served as a pre-treatment assessment in this study. Eleven out of 44 participants (25.0%; 23.3% ICBT and 28.6% IPDT) did not provide post-treatment data. At the 7-month follow-up, 4 out of 44 participants (9.1%; 6.7% ICBT and 14.3% IPDT) did not provide self-report data. Four participants from the ICBT group and two from the IPDT group were unreachable for the telephone interview and were in accordance with the intention-to-treat principle classified as unimproved on the CGI-I.

The number of completed treatment modules was used as a measure of adherence. In the psychodynamic group, a module was considered to be finished only if the weekly discussion of the module was sent to the therapist. Similarly, in the ICBT group, the module was classified as finished when the homework had been completed. Mean percentage of completed modules was 62.0% (SD = 33.2%) in the ICBT group and 73.8% (SD = 35.8%) in the IPDT group. This difference was not significant (*t*(42) = 1.07, *p* = .29). However, there were more participants who completed the whole program in the IPDT group (57.1%) than in the ICBT group (26.7%), *χ*^*2*^(*N* = 44, *df* = 1) = 3.83, *p* = .05.

### Primary outcome measure

Mixed-effects model analyses showed that both groups improved on the BDI-II from pre-treatment to post-treatment (*F*(1, 253.0) = 100.6, *p* < .001, for the ICBT group and *F*(1, 117.1) = 42.0, *p* < .001, for the IPDT group). This is mirrored by within-group effect sizes on the BDI-II around *d* = 1.0 in both groups, as seen in Table 2. No significant further effects of time were found within the groups from post-treatment to follow-up. There were no significant interaction effects of group and time, neither from pre to post, nor pre to follow-up (both *F*’s < 0.37, both *p*’s > .55).

**Table 2:** **Descriptive statistics of means, SDs and effect sizes (Cohen’s *d*) for measures of depression, anxiety and quality of life**

		**Mean (SD)**				**Effect size *d *(95% CI)**			
**Outcome measure**	**Group**	**Pre ctrl**	**Post ctrl/Pre tx**	**Post tx**	**7-month follow-up**	**Between-group, Post tx**	**Between-group, 7mo**	**Within-group, Pre tx – Post tx**	**Within-group, Post tx - 7mo**
BDI-II									
	ICBT	26.10 (6.7)	19.57 (8.0)	12.26 (6.6)	10.36 (10.6)	0.33 (−0.42 – 1.07)	0.45 (−0.23 – 1.14)	1.04 (0.61 – 1.48)	0.20 (−0.10 – 0.50)
	IPDT	26.29 (6.8)	22.29 (7.4)	14.60 (8.5)	15.42 (12.6)			1.06 (0.45 – 1.67)	−0.13 (−0.69 – 0.43)
MADRS-S									
	ICBT	24.17 (4.6)	18.30 (6.8)	12.78 (7.8)	11.11 (8.2)	0.28 (−0.46 – 1.03)	0.32 (−0.36 – 1.00)	0.88 (0.43 – 1.34)	0.18 (−0.13 – 0.49)
	IPDT	22.64 (5.9)	19.93 (5.4)	14.90 (6.7)	13.75 (8.4)			0.90 (0.21 – 1.58)	0.00 (−0.44 – 0.44)
PHQ-9									
	ICBT	13.03 (4.0)	10.63 (5.1)	6.17 (5.1)	5.75 (6.0)	0.11 (−0.63 – 0.85)	0.36 (−0.32 – 1.04)	1.02 (0.46 – 1.57)	0.19 (−0.12 – 0.50)
	IPDT	14.14 (5.1)	11.93 (3.9)	6.70 (4.3)	7.92 (6.4)			1.08 (0.65 – 1.51)	−0.21 (−0.56 – 0.15)
GAD-7									
	ICBT	8.60 (3.8)	7.33 (4.8)	5.09 (4.3)	3.71 (4.9)	0.14 (−0.61 – 0.88)	0.70 (0.00 – 1.39)	0.56 (0.15 – 0.97)	0.27 (0.01 – 0.53)
	IPDT	7.57 (4.7)	8.71 (5.2)	5.70 (4.9)	7.08 (4.8)			0.63 (−0.05 – 1.31)	−0.45 (−1.07 – 0.17)
BAI									
	ICBT	19.13 (10.0)	12.67 (8.6)	9.43 (8.1)	7.93 (9.9)	0.22 (−0.53 – 0.96)	0.38 (−0.30 – 1.06)	0.39 (0.04 – 0.75)	0.09 (−0.24 – 0.42)
	IPDT	18.07 (9.7)	15.29 (9.9)	11.10 (6.6)	11.83 (11.0)			0.61 (−0.13 – 1.35)	−0.23 (−0.74 – 0.28)
QOLI									
	ICBT	−0.07 (1.1)	0.15 (1.5)	0.58 (1.6)	1.49 (1.7)	0.32 (−0.43 – 1.07)	0.67 (−0.02 – 1.36)	0.45 (0.06 – 0.83)	0.45 (0.13 – 0.78)
	IPDT	−0.19 (1.6)	0.33 (1.4)	0.09 (1.2)	0.46 (1.1)			0.22 (−0.14 – 0.57)	−0.20 (−0.56 – 0.16)

*Note*: Pre ctrl = Time point before original trial started; Post ctrl/Pre tx = Time point at the end of the original trial period which also served as pre-treatment assessment in this study; Post tx = Time point at the end of the treatment period; BDI-II = Beck Depression Inventory-II; MADRS-S = Montgomery Åsberg Depression Rating Scale - Self Rated; PHQ-9 = 9-item Patient Health Questionnaire Depression Scale; GAD-7 = 7-item Patient Health Questionnaire Generalized Anxiety Disorder Scale; BAI = Beck Anxiety Inventory; QOLI = Quality of Life Inventory; ICBT = Internet-based cognitive-behavioral therapy; IPDT = Internet-based psychodynamic therapy.

### Secondary outcome measures

For secondary measures of depression (the MADRS-S and the PHQ-9), random intercept models showed significant effects of time in both groups, from pre-treatment to post-treatment (all *F*’s > 10.3, all *p*’s < .01). No significant interaction effects of group and time were observed - neither on the MADRS-S nor the PHQ-9.

For other secondary measures (GAD-7, BAI and QOLI), the mixed models-analyses showed significant effects of time from pre-treatment to post-treatment in the CBT group (all *F*’s > 4.51, all *p*’s < .05). No effects of time were found in the IPDT group for the BAI or the QOLI (both *F*’s < 2.97, both *p*’s > .12). A borderline significant effect of time, from pre-treatment to post-treatment, was found in the IPDT group for the GAD-7 (*F*(1, 10.3) = 4.47, *p* = .06).

Random intercept models revealed a significant interaction effect of group and time on the QOLI, from pre-treatment to follow-up, favoring ICBT. A post-hoc *t*-test comparing the two groups at follow-up was marginally significant, *t*(38) = 1.94, *p* = .06. No other interaction effects of group and time were found for secondary measures.

From post-treatment to follow-up, there were significant effects of time in the ICBT group for the GAD-7 (*F*(1, 24.1) = 4.89, *p* < .05) and the QOLI (*F*(1, 24.6) = 10.0, *p* < .01). The corresponding within-group effect sizes were *d* = 0.27 (95% CI: 0.01 – 0.53) for the GAD-7 and *d* = 0.45 (95% CI: 0.13 – 0.78) for the QOLI, indicating small but significant improvements after treatment termination.

### Recovery after treatment

There were no differences in recovery rates (post-treatment BDI-II score of ≤ 10) between groups, neither at post-treatment (IPDT: *n* = 5, 35.7%; ICBT: *n* = 9, 30.0%), *χ*^*2*^(*N* = 44, *df* = 1) = 0.14, *p* = 0.71, nor at follow-up (IPDT: *n* = 5, 35.7%; ICBT: *n* = 18, 60.0%), *χ*^*2*^(*N* = 44, *df* = 1) = 2.25, *p* = 0.13. However, the number of participants in the ICBT group who recovered doubled from 30% at post-treatment to 60% at follow-up. A separate analysis of the ICBT group revealed a significant increase of recoverers from post-treatment to follow-up (McNemar’s *χ*^*2*^(*N* = 30, *df* = 1) = 9.00, *p* < .01).

### Preference and predicting treatment outcome and adherence

The strength of preference was 2.90 (SD: 0.85) and 2.93 (SD: 1.07) in the ICBT group (*N* = 30) and the IPDT group (*N* = 14), respectively. No significant difference in this variable was observed (*t*(42) = 0.096, *p* = .92).

The prediction data regarding outcome for the two groups on the BDI-II are presented in Table 3. For the entire sample and for the IPDT group separately, strength of preference was not correlated with treatment outcome, neither at post-treatment nor at follow-up. However, in the ICBT group, preference ratings predicted outcome at the follow-up (*r* = −.42, *p* < .05).

**Table 3:** **Correlations (Pearson's *r*) between strength of preference and outcome, adherence and completion of the entire program**

	**BDI-II post**	**BDI-II follow-up**	**Adherence**	**Completion**
ICBT	-.13	-.42*	.30	.16
IPDT	.19	.42	.24	.50†
Total	-.01	-.13	.28†	.29†

*Note*: †*p* < .10 *p < .05. ICBT = Internet-based cognitive-behavioral therapy; IPDT = Internet-based psychodynamic therapy; BDI-II = Beck Depression Inventory-II.

In the entire sample, there was a close to significant correlation between strength of preference and adherence to treatment (*r* = .28, *p* = .07). This correlation was not significant in any subgroup analyses. For the IPDT group and for the entire sample, there were trends towards significant correlations between strength of preference and completion of the treatment program (*r* = .50, *p* = .07 and *r* = .29, *p* = .06, respectively).

### Clinical global impression and adverse events

There were no differences between groups in degree of improvement according to the CGI-I, *χ*^*2*^(*N* = 44, *df* = 1) = 0.42, *p* = 0.52. In the ICBT group and IPDT group respectively, there were 16 and 6 participants (53.3% and 42.9%) who were much or very much improved.

Two participants from the ICBT group were “minimally worse” and “very much worse” as assessed by the CGI-I, and were therefore classified as adverse events. Both these participants stated change in medication as the reason for feeling worse. An additional adverse event was noted as one participant from the psychodynamic treatment group was “much worse”. Reason for this was not clearly stated by the participant.

## Discussion

In this pilot study, we investigated differences between psychodynamic and cognitive-behavioral psychotherapy delivered via the Internet in a sample of participants with major depression, where groups were formed based on preference. We also investigated the predictive value of the strength of preference. The main finding regarding efficacy was that there were indications that those choosing ICBT may have had some larger long-term benefits in terms of quality of life. Another finding was that completion of the entire program was higher among participants who chose the psychodynamic treatment. We also found indications that strength of preference could predict adherence. Among participants choosing psychodynamic treatment, completion of the program was predicted by strength of preference. Moreover, participants preferred ICBT over IPDT as significantly more participants chose ICBT.

Despite a significantly larger rate of completion in the psychodynamic treatment group, we found no indications of differential efficacy during the treatment phase. However, indications of treatment differences were found at the 7-month follow-up in favor of ICBT. This is somewhat in line with results which show that participants having received ICBT may benefit from it 3.5 years after completion [33]. It is important to notice that all participants who did not complete the entire treatment, did receive the remaining modules after post-measurement. As data on post-treatment use of treatment modules were not available, we could not perform any analyses to investigate if continued work with the modules were related to continued symptom reduction. While the two programs had a similar amount of text, the ICBT treatment had homework in the classical sense (completing exercises) and the IPDT treatment had encouragements for working with the material. The work that the ICBT group was doing was known to the participants as “homework” while it in the IPDT group was called “reflections”. The latter was more of writing about experiences than completing tasks. This could have made a difference for how the participants worked with the material and it could potentially be related to how many participants completed the entire treatment. While we in this study did not measure the amount of work done by each participant, this could potentially also have been affected by this difference in how the work was presented to and handled by the participants.

This study also explored preferences, as all participants had been allocated to their preferred choice of treatment and had stated their strength of preference. The preference literature suggest that an extra effect of having received one’s preferred treatment should be expected. This study can only speculate about this, as all preferences were matched. However, the within-group effect size on the primary outcome measure in this study was around *d* = 1.0, which seems quite low when compared to *d* = 2.18 from the original trial [9]. Importantly, all participants from this study had previously taken part of a support intervention for 10 weeks, with substantial within-group effects. Still, based on this comparison, data from this pilot study do not seem to give any support for an extra effect of preference matching. Importantly, the effect of preference matching seems to be less important when comparing two psychotherapies, than when comparing psychotherapy and pharmacotherapy [12].

We also considered preference strength in this study. Our indications that strength of preference could predict adherence and completion of treatment are similar to the results presented by Raue et al. [13]. In contrast to the two previous studies [13,14] we found that strength of preference could predict long-term treatment outcome in the ICBT group. In summary, results regarding preference strength are mixed. However, this study indicates that strength of preference for treatment has some predictive value and that further research is warranted.

There are limitations to this study that need to be addressed. First, as one of the aims was to explore differences in efficacy between treatments, the study is underpowered. If this question is to be further explored, a larger sample needs to be used. Second, as all participants received their preferred treatment, we could only explore the effect of preference matching in an open study design. A preferable design would be to randomize participants to either a preferred or non preferred treatment arm [34]. Third, we did not explore therapist preferences, i.e. allegiance effects. We do not rule out that therapist preferences could have potentially made impact in this study and acknowledge the effect of allegiance as an important area for further research. Fourth, we did not measure factors that could potentially explain differences in completion rates, e.g. treatment satisfaction and perceived effort of taking part of treatment.

The findings from this pilot study suggest several research issues. A larger, adequately powered, randomized trial comparing the two treatments head-to-head could investigate differences in efficacy and the predictive value of preference strength regarding treatment outcome and adherence to treatment. If designed as a four-armed trial (randomized to being given the choice or not, and if not, randomized to treatment arm), as described by Howard and Thornicroft [34], the trial could also investigate the preference matching effect. This design would be a solution to all of the limitations mentioned above. Furthermore, as the effect of preference strength are largely unexplored, one implication of this study is that we have shown that strength of preference indeed may have some predictive value and further inclusion of this in treatment studies is warranted. Research on the effect of treatment preference and strength of preference could potentially be useful in dissemination of Internet-delivered psychological treatments, where these factors could be taken into consideration.

## Conclusions

This exploratory study shows that there may be differences between psychodynamic and cognitive-behavioral Internet-delivered psychotherapy. IPDT may lead to more participants completing the treatment and ICBT may have larger long-term benefits. More participants preferred ICBT, but strength of preference was equally distributed across the two groups. The study indicates that strength of preference has a predictive value and should be included in future studies investigating patients’ preferences.

## Competing interests

The authors declared that they have no competing interests.

## Authors’ contributions

RJ in collaboration with GA designed the study. AN collected all data from the follow-up. RJ and AN made the data analyses. RJ drafted the manuscript. P Carlbring, P Cuijpers, AN and GA reviewed and revised the manuscript. All authors have read and approved the final manuscript to be published.

## Pre-publication history

The pre-publication history for this paper can be accessed here:

http://www.biomedcentral.com/1471-244X/13/268/prepub

## References

[B1] Ebmeier KP Donaghey C Steele JD Recent developments and current controversies in depression Lancet 2006 367 153 167 10.1016/S0140-6736(06)67964-6 16413879

[B2] Smit F Cuijpers P Oostenbrink J Batelaan N de Graaf R Beekman A Costs of nine common mental disorders: implications for curative and preventive psychiatry J Ment Health Policy Econ 2006 9 193 200 17200596

[B3] Cuijpers P van Straten A Andersson G van Oppen P Psychotherapy for depression in adults: a meta-analysis of comparative outcome studies J Consult Clin Psychol 2008 76 909 922 19045960 10.1037/a0013075

[B4] Driessen E Cuijpers P de Maat SCM Abbass AA de Jonghe F Dekker JJM The efficacy of short-term psychodynamic psychotherapy for depression: a meta-analysis Clin Psychol Rev 2010 30 25 36 10.1016/j.cpr.2009.08.010 19766369

[B5] Andersson G Using the Internet to provide cognitive behaviour therapy Behav Res Ther 2009 47 175 180 10.1016/j.brat.2009.01.010 19230862

[B6] Johansson R Andersson G Internet-based psychological treatments for depression Expert Rev Neurother 2012 12 861 870 10.1586/ern.12.63 22853793

[B7] Andrews G Cuijpers P Craske MG McEvoy P Titov N Computer therapy for the anxiety and depressive disorders is effective, acceptable and practical health care: a meta-analysis PLoS ONE 2010 5 e13196 10.1371/journal.pone.0013196 20967242 PMC2954140

[B8] Cuijpers P Donker T van Straten A Li J Andersson G Is guided self-help as effective as face-to-face psychotherapy for depression and anxiety disorders? A systematic review and meta-analysis of comparative outcome studies Psychol Med 2010 40 1943 1957 10.1017/S0033291710000772 20406528

[B9] Johansson R Ekbladh S Hebert A Lindström M Möller S Petitt E Poysti S Larsson MH Rousseau A Carlbring P Cuijpers P Andersson G Psychodynamic guided self-help for adult depression through the internet: a randomised controlled trial PLoS ONE 2012 7 e38021 10.1371/journal.pone.0038021 22741027 PMC3362510

[B10] Andersson G Paxling B Roch-Norlund P Östman G Norgren A Almlöv J Georén L Breitholtz E Dahlin M Cuijpers P Carlbring P Silverberg F Internet-based psychodynamic vs. cognitive behavioural guided self-help for generalized anxiety disorder: A randomised controlled trial Psychother Psychosom 2012 81 344 355 10.1159/000339371 22964540

[B11] Swift JK Callahan JL The impact of client treatment preferences on outcome: a meta-analysis J Clin Psychol 2009 65 368 381 10.1002/jclp.20553 19226606

[B12] Swift JK Callahan JL Vollmer BM Preferences J Clin Psychol 2011 67 155 165 10.1002/jclp.20759 21120917

[B13] Raue PJ Schulberg HC Heo M Klimstra S Bruce ML Patients’ depression treatment preferences and initiation, adherence, and outcome: a randomized primary care study Psychiatr serv 2009 60 337 343 10.1176/appi.ps.60.3.337 19252046 PMC2710876

[B14] Dunlop BW Kelley ME Mletzko TC Velasquez CM Craighead WE Mayberg HS Depression beliefs, treatment preference, and outcomes in a randomized trial for major depressive disorder J Psychiatr Res 2012 46 375 381 10.1016/j.jpsychires.2011.11.003 22118808 PMC3288535

[B15] Andersson G Estling F Jakobsson E Cuijpers P Carlbring P Can the patient decide which modules to endorse? An open trial of tailored internet treatment of anxiety disorders Cogn Behav Ther 2011 40 57 64 10.1080/16506073.2010.529457 21337215

[B16] Andersson G Bergström J Holländare F Carlbring P Kaldo V Ekselius L Internet-based self-help for depression: randomised controlled trial Br J Psychiatry 2005 187 456 461 10.1192/bjp.187.5.456 16260822

[B17] Svanborg P Åsberg M A new self-rating scale for depression and anxiety states based on the Comprehensive Psychopathological Rating Scale Acta Psychiatr Scand 1994 89 21 28 8140903 10.1111/j.1600-0447.1994.tb01480.x

[B18] Kroenke K Spitzer RL Williams JB The PHQ-9: validity of a brief depression severity measure J Gen Intern Med 2001 16 606 613 10.1046/j.1525-1497.2001.016009606.x 11556941 PMC1495268

[B19] Beck AT Epstein N Brown G Steer RA An inventory for measuring clinical anxiety: psychometric properties J Consult Clin Psychol 1988 56 893 897 3204199 10.1037//0022-006x.56.6.893

[B20] Spitzer RL Kroenke K Williams JBW Löwe B A brief measure for assessing generalized anxiety disorder: the GAD-7 Arch Intern Med 2006 166 1092 1097 10.1001/archinte.166.10.1092 16717171

[B21] Frisch M Cornell J Villanueva M Retzlaff P Clinical validation of the Quality of Life Inventory: A measure of life satisfaction for use in treatment planning and outcome assessment Psychol Assess 1992 4 92 101

[B22] Holländare F Andersson G Engström I A comparison of psychometric properties between internet and paper versions of two depression instruments (BDI-II and MADRS-S) administered to clinic patients J Med Internet Res 2010 12 e49 10.2196/jmir.1392 21169165 PMC3057311

[B23] Carlbring P Brunt S Bohman S Austin D Richards J Öst L-G Andersson G Internet vs. paper and pencil administration of questionnaires commonly used in panic/agoraphobia research Comput Hum Behav 2007 23 1421 1434 10.1016/j.chb.2005.05.002

[B24] Guy W Clinical Global Impressions. ECDEU Assessment Manual for Psychopharmacology 1976 Rockville: NIMH

[B25] Silverberg F Make the Leap: A Practical Guide to Breaking the Patterns that Hold You Back 2005 New York: Marlowe & Co

[B26] Vernmark K Lenndin J Bjärehed J Carlsson M Karlsson J Öberg J Carlbring P Eriksson T Andersson G Internet administered guided self-help versus individualized e-mail therapy: A randomized trial of two versions of CBT for major depression Behav Res Ther 2010 48 368 376 10.1016/j.brat.2010.01.005 20152960

[B27] Gueorguieva R Krystal JH Move over ANOVA: progress in analyzing repeated-measures data and its reflection in papers published in the Archives of General Psychiatry Arch Gen Psychiat 2004 61 310 317 10.1001/archpsyc.61.3.310 14993119

[B28] Steketee G Chambless D Methodological issues in prediction of treatment outcome Clin Psychol Rev 1992 12 387 400 10.1016/0272-7358(92)90123-P

[B29] Dimidjian S Hollon SD Dobson KS Schmaling KB Kohlenberg RJ Addis ME Gallop R McGlinchey JB Markley DK Gollan JK Atkins DC Dunner DL Jacobson NS Randomized trial of behavioral activation, cognitive therapy, and antidepressant medication in the acute treatment of adults with major depression J Consult Clin Psychol 2006 74 658 670 16881773 10.1037/0022-006X.74.4.658

[B30] Kessler D Lewis G Kaur S Wiles N King M Weich S Sharp DJ Araya R Hollinghurst S Peters TJ Therapist-delivered Internet psychotherapy for depression in primary care: a randomised controlled trial Lancet 2009 374 628 634 10.1016/S0140-6736(09)61257-5 19700005

[B31] Borenstein M Hedges LV Higgins JPT Rothstein HR Introduction to Meta-Analysis 2009 Oxford: John Wiley & Sons

[B32] Cohen J Statistical Power Analysis for the Behavioral Sciences 1988 2 Hillsdale, N.J.: Lawrence Erlbaum Associates

[B33] Andersson G Hesser H Hummerdal D Bergman-Nordgren L Carlbring P A 3.5-year follow-up of Internet-delivered cognitive behavior therapy for major depression J Ment Health 2011 2011 2011 10.3109/09638237.2011.608747 21957933

[B34] Howard L Thornicroft G Patient preference randomised controlled trials in mental health research Br J Psychiatry 2006 188 303 304 10.1192/bjp.188.4.303 16582054

